# Rare detection of dermal *Leishmania infantum* in two pediatric patients with cutaneous leishmaniasis (CL) in southern Iran

**DOI:** 10.1016/j.parepi.2025.e00452

**Published:** 2025-08-02

**Authors:** Mohsen Kalantari, Kourosh Azizi, Qasem Asgari, Masoud Yousefi

**Affiliations:** a Research Center for Health Sciences, Institute of Health, Department of Medical Entomology and Vector Control, School of Health, Shiraz University of Medical Sciences, Shiraz, Iran; b Department of Medical Parasitology and Mycology, School of Medicine, Shiraz University of Medical Sciences, Shiraz, Iran; c Department of Environmental Health Engineering, Mamasani Higher Education Complex for Health, Shiraz University of Medical Sciences, Shiraz, Iran

**Keywords:** Cutaneous leishmaniasis, Visceral leishmaniasis, Epidemiology, PCR, Iran

## Abstract

This study highlights the rare detection of dermal *Leishmania infantum* in two pediatric patients with cutaneous leishmaniasis (CL) in southern Iran. Both patients had a history of visceral leishmaniasis (VL) and presented with atypical facial lesions. Molecular assays confirmed the presence of L. *infantum* through species PCR targeting the cysteine protease B (cpb) gene, revealing 702/741-bp amplicons. Phylogenetic analysis describes two pediatric CL cases in southern Iran caused by a strain of L. *infantum* closely related to the LIPA59 genotype. This detection in immunocompetent children challenges the traditional association of L. *infantum* solely with VL and highlights its capacity for atypical dermal manifestations. These findings underscore the necessity of molecular diagnostics to differentiate *Leishmania* species, as misidentification risks ineffective treatments and potential disease progression. Enhanced surveillance integrating PCR-based methods is critical to track dermal strains in regions where VL and CL overlap, particularly given potential ecological or genetic drivers of atypical tropism. Addressing this emerging threat requires integrated strategies to mitigate the dual burden of cutaneous and visceral disease in vulnerable populations.

## Highlights

• Molecular confirmation of dermal *Leishmania infantum* causing cutaneous leishmaniasis (CL) in immunocompetent pediatric patients in southern Iran.
• Both patients had prior visceral leishmaniasis, suggesting parasite persistence or reactivation in dermal tissues despite systemic clearance.
• Phylogenetic analysis revealed genetic similarity to Mediterranean strains, implicating regional transmission or adaptive evolution.
• Public health implications include risks of treatment failure and persistent transmission due to misdiagnosis
• Atypical CL presentations challenge emphasizing the need for PCR diagnostics to prevent misdiagnosis and ineffective treatment.

## 1 Introduction

Leishmaniasis, a vector-borne disease caused by protozoan parasites of the *Leishmania* genus, manifests in three primary clinical forms: visceral (VL), cutaneous (CL), and mucocutaneous leishmaniasis (MCL) (Tao and Jia, 2024). Among these, VL, often fatal if untreated, is predominantly caused by *Leishmania infantum* and L. *donovani* in the Old World and *Leishmania chagasi* in the New World, transmitted through the bite of infected female phlebotomine sand flies (Arumugam et al., 2022). In contrast, CL, characterized by localized skin lesions, is typically associated with species such as *L. major* and *L. tropica*. However, emerging evidence suggests that L. *infantum*, traditionally linked to systemic visceral infections, may also exhibit dermal behavior, leading to atypical cutaneous presentations (Ekemen et al., 2024). These cases are sporadically documented, primarily in immunocompromised individuals, such as those with HIV coinfection or undergoing immunosuppressive therapies; PKDL, a dermal sequela typically associated with L. *donovani* infection, is rarely linked to L. *infantum* in conjunction with post-kala-azar dermal leishmaniasis (PKDL) or in immunocompromised individuals, such as those with HIV coinfection or undergoing immunosuppressive therapies (Monge-Maillo et al., 2022).

Globally, *L. infantum* is endemic in Mediterranean regions, South America, and parts of Asia, with Iran representing a significant focus due to its diverse ecological niches supporting sand fly vectors. In Iran, VL is hyperendemic in the southern provinces (e.g., Fars, Hormozgan) and northwestern regions (e.g., Ardabil), where L. *infantum* is primarily transmitted by *Phlebotomus kandelakii* and *P. perfiliewi*. Despite this, CL in the country is overwhelmingly attributed to L. *major* (in arid zones) and L. *tropica* (in urban areas), with L. *infantum* rarely implicated in skin-specific pathology (Ghatee et al., 2020). The scarcity of reported L. *infantum*-CL cases raises questions about parasite strain variability, host immune responses, and environmental factors influencing tropism.

Early molecular studies identified genetically distinct strains of L. *infantum*, such as the dermal LIPA59 genotype, which exhibit unique adaptations to cutaneous tissues (Gritti et al., 2025; Hide and Banuls, 2006). Subsequently, a substantial body of literature has documented L. *infantum*-CL cases across Mediterranean regions, South America, and the Middle East, indicating broader epidemiological relevance than previously recognized. These strains challenge the conventional dichotomy of *Leishmania* species and their clinical associations, suggesting a continuum of pathogenicity influenced by genomic plasticity (Arumugam et al., 2022; Dantas-Torres, 2024; Ekemen et al., 2024). In immunocompetent hosts, such strains may evade systemic dissemination, instead persisting in dermal macrophages and provoking localized immune reactions (Quaresma, 2019). This phenomenon underscores the need for advanced diagnostic methods, including PCR-based genotyping, to differentiate species and strains, as misidentification can lead to inappropriate therapeutic strategies (Marlowe and Wolk, 2008).

The emergence of L. *infantum*-CL in non-immunocompromised populations, particularly children, is a growing public health concern. Pediatric cases are especially critical, as delayed diagnosis or mismanagement may result in chronic disfigurement or inadvertent progression to visceral involvement (Singh et al., 2023). In southern Iran, where VL is entrenched, the detection of CL caused by L. *infantum* highlights evolving epidemiological patterns, potentially driven by ecological changes, vector adaptation, or human migration (Asgari et al., 2020). This report describes two pediatric CL cases in southern Iran caused by a strain of L. *infantum* closely related to the LIPA59 genotype, emphasizing the parasite's expanding clinical repertoire and the necessity for heightened surveillance in endemic zones. Understanding these atypical presentations is vital for refining diagnostic protocols, guiding treatment, and mitigating the neglected tropical disease burden in vulnerable populations.

## 2 Methods

### 2.1 Study setting and participants

Between 2018 and 2020, 354 suspected cutaneous leishmaniasis (CL) cases from Shiraz and its suburbs in Fars province, southern Iran, were screened. Initial diagnosis was performed by microscopic examination of lesion smears and culture. Two pediatric patients with a history of visceral leishmaniasis (VL) and presenting with atypical facial lesions were the focus of this report.

### 2.2 Molecular diagnostics

DNA was extracted from lesion smears and cultured promastigotes. Two sequential PCR assays were performed: first, a screening PCR targeting of the minicircle kinetoplast DNA (kDNA) to detect *Leishmania* genus (Asgari et al., 2020), and second, a species-specific PCR targeting the cysteine protease B (*cpb*) gene to differentiate *Leishmania infantum* from other species (Hide and Banuls, 2006). Positive PCR products were sequenced for confirmation.

### 2.3 Phylogenetic analysis

The obtained sequences from the *cpb* gene PCR (approximately 702 bp) were aligned with reference strains from GenBank using MUSCLE in MEGA-7. Phylogenetic trees were constructed using both Neighbor-Joining and Maximum Likelihood methods, with bootstrap analysis of 1000 replicates (Zhang et al., 2013).

## 3 Results

### 3.1 Epidemiological findings

Among the 276 confirmed CL cases in southern Iran, the highest infection rate (56.9 %) occurred in individuals aged 11–30 years, with an equal gender distribution (138 males and 138 females).

#### 3.1.1 Case reports

•
  Case 1 A 4-year-old male with a history of VL hospitalization (treated one years prior with liposomal amphotericin B: 3 mg/kg/day for 10 days) presented with a 2-month-old ulcerative lesion on the left cheek. Microscopy revealed amastigotes, and culture isolated promastigotes. PCR confirmed L. infantum with a *cpb* amplicon (702/741-bp) characteristic of strains closely related to the LIPA59 genotype. The lesion was treated with intralesional meglumine antimoniate (0.5 mL/cm^2^, weekly for 6 weeks), achieving complete re-epithelialization after 8 weeks. No recurrence was observed during 6 months of follow-up.
•
  Case 2 A 7-year-old male, also with prior VL (treated two years prior with liposomal amphotericin B: identical regimen), exhibited a similar facial lesion. Molecular assays identified the same *cpb* profile, indicative of close similarity to the LIPA59 genotype. Systemic meglumine antimoniate (20 mg/kg/day for 21 days) was administered, with lesion resolution at 12 weeks. Mild transient arthralgia occurred during treatment but resolved without intervention. No relapse was documented over 9 months.

Both cases lacked HIV co-infection or other immunodeficiency. Lesions were open and ulcerative, contrasting with nodular PKDL patterns seen in India.

### 3.2 Molecular and phylogenetic results

**Fig. 1:**
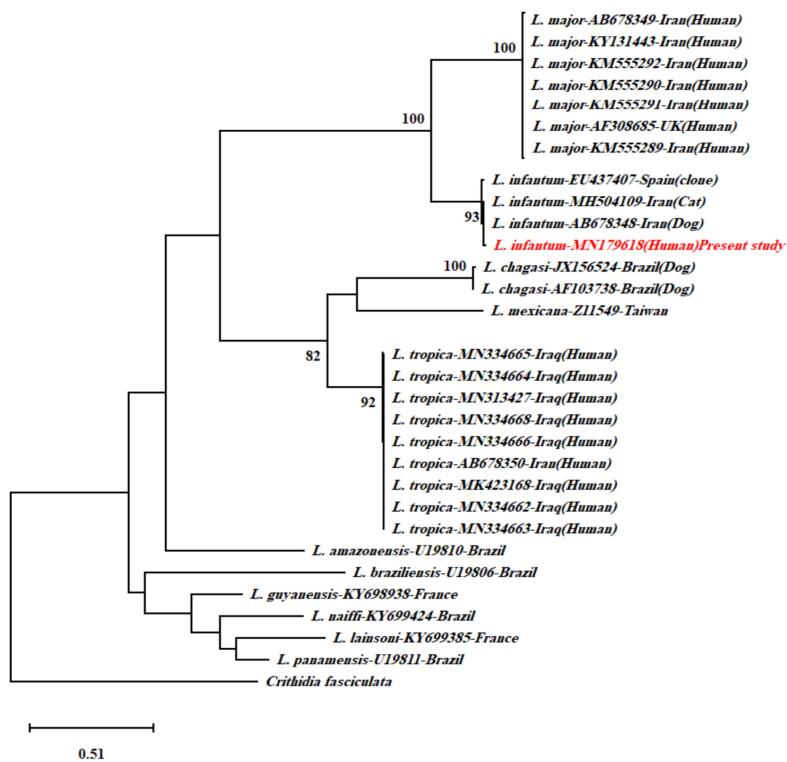
Phylogenetic tree of *Leishmania* strains based on partial *cpb* gene sequences (702 bp)**.** Bootstrap values (1000 replicates) are shown. Iranian isolates cluster with Mediterranean strains (Spain EU437407; Iran AB678348).

The L. *infantum* isolates from both patients were confirmed by PCR and sequencing of the *cpb* gene, showed 702/741-bp amplicons consistent with strains phylogenetically related to the LIPA59 genotype described by Hide and Banuls (2006)**.** Phylogenetic analysis of the partial ***cpb*** gene sequences (702 bp) revealed that the isolates clustered closely with strains from Spain (EU437407) and Iran (AB678348) (Fig. 1). This genetic homogeneity suggests regional transmission networks or historical introductions from VL-endemic areas.

## 4 Discussion

The detection of dermal *Leishmania infantum* in immunocompetent pediatric patients with prior visceral leishmaniasis (VL) challenges established epidemiological and clinical paradigms. Typically, *L. infantum* is associated with systemic infections, yet its emergence as a causative agent of cutaneous leishmaniasis (CL) in southern Iran underscores the parasite's genomic plasticity and adaptive potential. These cases align with sporadic reports from Mediterranean regions and South America, where atypical L. *infantum* strains have been implicated in CL, particularly in individuals with compromised immunity or PKDL (Arumugam et al., 2022; Dantas-Torres, 2024). However, the absence of HIV coinfection or immunosuppressive therapies in these pediatric cases suggests that strain-specific adaptations, rather than host vulnerability alone, may drive cutaneous tropism. Genetic analyses of these isolates revealed close phylogenetic clustering with Mediterranean isolates (e.g., Spain's EU437407), which may suggest historical introductions via human migration or reservoir host movement. Such cross-border transmission dynamics are further supported by studies in Sudan and India, where hybrid *Leishmania* strains complicate control efforts due to their dual visceral and cutaneous pathogenicity (Singh et al., 2023).

The patients' prior VL history raises critical questions about parasite persistence and reactivation mechanisms. Residual L. *infantum* parasites may evade systemic clearance by infiltrating dermal macrophages, where they establish latent infections (Soares et al., 2024). Immunological studies suggest that partial immunity post-VL could suppress visceral dissemination but fail to eradicate dermal reservoirs, enabling localized reactivation under stressors such as malnutrition or co-infections (Monge-Maillo et al., 2022). Alternatively, genetic mutations in L. *infantum* strains with dermal tropism**,** particularly in virulence factors like cysteine protease B (*cpb*), may enhance cutaneous tissue affinity. Comparative genomic studies of such dermal strains have identified polymorphisms in *cpb* and other surface proteins that alter host-cell interactions, potentially favoring dermal macrophage colonization over visceral organ targeting (Hide and Banuls, 2006). In our isolates, the dual-band PCR profile (702/741-bp) in the ***cpb*** gene suggests heterozygosity, a feature documented in the LIPA59 dermotropic strain by Hide and Banuls (2006). This evolutionary trajectory mirrors findings in Brazil, where L. *infantum* CL isolates exhibit unique lipophosphoglycan (LPG) profiles that influence vector midgut adhesion and skin-specific infectivity (Soares et al., 2024).

Environmental and ecological factors further amplify the risk of atypical L. *infantum* transmission. Climate change has expanded the geographic range of *Phlebotomus* vectors in Iran, while urbanization increases human exposure to peridomestic sand fly habitats (Ghatee et al., 2020). The genetic homogeneity between Iranian and Mediterranean strains suggests that vector competence may also play a role; *P. perfiliewi***,** a primary VL vector in Iran, could exhibit altered feeding behaviors or salivary gland interactions that favor cutaneous parasite retention (Rassi et al., 2024).

Similarly, reservoir host dynamics—particularly the role of domestic dogs—may require re-evaluation based on studies suggesting that dogs with subclinical L. *infantum* infections act as persistent reservoirs, and xenodiagnostic evidence indicates dermal strains disproportionately colonize canine skin, potentially increasing zoonotic transmission risk (Scorza et al., 2021; Ribeiro et al., 2024).

The clinical and public health implications of these findings are profound. Misdiagnosis of L. *infantum*-CL as L. *major* or L. *tropica* infections could lead to inappropriate therapies, as antimonial drugs show variable efficacy against dermal strains. This misdiagnosis risks treatment failure, chronic lesions, and potential progression to visceral involvement, especially in pediatric populations. Furthermore, mismanagement may contribute to the emergence of drug resistance and increased transmission due to persistent infectious reservoirs. Moreover, untreated cutaneous cases may act as cryptic reservoirs, perpetuating transmission cycles. Integrated molecular surveillance, combining PCR-based genotyping and phylogenetic tools, is critical for tracking strain diversity and informing targeted interventions (de Vries and Schallig, 2022). In Iran, expanding surveillance to VL-endemic regions with overlapping CL incidence—such as Fars and Hormozgan provinces—could elucidate transmission hotspots and strain spillover mechanisms.

The rarity of reported dermal L. *infantum* CL cases in Iran, highlighted in this study, raises significant concerns about underdiagnosis and national reporting accuracy. Cutaneous leishmaniasis (CL) is a notifiable disease in Iran, mandated by the national surveillance system. However, reporting primarily relies on clinical presentation and microscopic confirmation of Leishmania amastigotes in lesion samples, techniques that cannot differentiate L. *infantum* from L. *major* or L. *tropica*. Consequently, cases caused by L. *infantum* are highly likely misclassified within the broader CL statistics, typically attributed to the more common species endemic in the region. This systemic lack of routine molecular surveillance using species-specific PCR or other genotyping methods has inevitably led to substantial underreporting of dermal L. *infantum* infections. The absence of accurate species identification in national data obscures the true prevalence and geographical distribution of this atypical manifestation, hindering risk assessment, targeted resource allocation, and the development of specific control strategies. Our findings underscore that the perceived rarity of L. *infantum*-CL may largely be an artifact of diagnostic limitations within the current surveillance framework rather than true epidemiological scarcity (Sharifi et al., 2023).

Finally, these cases highlight gaps in understanding host-parasite-vector interactions. The ulcerative morphology of lesions in these patients contrasts with nodular PKDL patterns, suggesting distinct immune pathways. Histopathological comparisons of L. *infantum*-CL and PKDL lesions could clarify whether parasite load, host cytokine profiles (e.g., IFN-γ, IL-10), or strain-specific factors drive phenotypic variation. Furthermore, experimental models using dermotropic L. *infantum* strains may unravel mechanisms of dermal persistence, such as metabolic adaptations to hypoxic skin environments or resistance to macrophage oxidative bursts.

## 5 Conclusion

This reports underscores L. *infantum*'s expanding clinical spectrum in Iran. The presence of dermal L. *infantum* in southern Iran reflects a convergence of genetic, environmental, and immunological factors. Addressing this threat requires a multidisciplinary approach, leveraging molecular epidemiology, vector ecology, and clinical research to mitigate the rising burden of atypical leishmaniasis. Furthermore, the integration of molecular diagnostics into public health surveillance systems is imperative to prevent misdiagnosis, optimize treatment, and interrupt transmission cycles in endemic regions.

## CRediT authorship contribution statement

**Mohsen Kalantari:** Writing – review & editing, Writing – original draft, Project administration, Conceptualization. **Kourosh Azizi:** Writing – review & editing, Validation, Supervision. **Qasem Asgari:** Writing – review & editing, Validation, Methodology. **Masoud Yousefi:** Writing – review & editing, Validation, Methodology.

## Ethical approval

This study was conducted in accordance with international, national, and institutional ethical guidelines. We declare that all experiments were performed in accordance with the ARRIVE guidelines 2.0 and that all experimental protocols were approved by Ethical approval obtained from the Science and Ethics Committee of Shiraz University of Medical Sciences (Approved ID: IR.SUMS.REC.1395.S475).

## Funding

Supported by 10.13039/501100004320Shiraz University of Medical Sciences (Grant: 94-01-104-10873).

## Declaration of competing interest

The authors of the present study declare no conflict of interests.

## Data Availability

Sequences deposited in GenBank (MN179618–MN179622).
